# Intraoperative Effect of a Bilateral Ultrasound-Guided Quadratus Lumborum Block in Cats Undergoing Ovariectomy

**DOI:** 10.3390/ani15050618

**Published:** 2025-02-20

**Authors:** José Diogo Dos-Santos, Mário Ginja, João Martins, Sofia Alves-Pimenta, Lénio Ribeiro, Pablo E. Otero, Bruno Colaço

**Affiliations:** 1 Research in Veterinary Medicine (I-MVET), Faculty of Veterinary Medicine, Lusófona University—Lisbon University Center, 1749-024 Lisbon, Portugal; 2 Veterinary and Animal Science Research Centre (CECAV), University of Trás-os-Montes e Alto Douro, (UTAD), 5000-801 Vila Real, Portugal; 3 Associate Laboratory for Animal and Veterinary Sciences (AL4AnimalS), 1300-477 Lisbon, Portugal; 4 Research Center for Biosciences and Health Technologies (CBIOS), Universidade Lusófona de Humanidades e Tecnologias, 1749-024 Lisbon, Portugal; 5 Department of Anesthesiology and Pain Management, Facultad de Ciencias Veterinarias, Universidad de Buenos Aires, Buenos Aires C1427CWN CABA, Argentina

**Keywords:** cat, ovariectomy, quadratus lumborum block, regional analgesia, sympathetic trunk block

## Abstract

Objective: Investigate the effect of bilateral ultrasound-guided quadratus lumborum block (QLB) in the need for additional opioid administration in response to intraoperative nociception during ovariectomy in cats. Methods: Thirty-six feral cats were randomly allocated to group control (C) or group QLB with 0.4 mL kg^−1^ of 0.25% bupivacaine per hemiabdomen. Hemodynamic data were collected at five intraoperative time points. A 20% increase in pulse rate or respiratory rate was treated with fentanyl. Results: Pulse rate and respiratory rate were higher in the C group compared to the QLB group during ovarian manipulation. No differences in systolic and mean arterial pressure and hypotension were comparable in both groups. The QLB group required less administration of intraoperative fentanyl than the C group at 4/16 and 10/16, respectively (*p* < 0.05). Conclusions: The bilateral QLB with bupivacaine led to a reduction in opioid consumption in feral cats undergoing elective ovariectomy.

## Simple Summary

Interfascial plane blocks have gained significant attention as a key approach for managing perioperative pain. However, there is limited research on the utilization of a bilateral ultrasound-guided quadratus lumborum block (QLB) on opioid consumption during intraoperative nociception in cats undergoing elective ovariectomy. This study used 32 feral cats that were randomly assigned to a control group (C) or a QLB group. The QLB group received 0.4 mL kg^−1^ of 0.25% bupivacaine per hemiabdomen. Hemodynamic parameters, including heart rate (HR) and respiratory rate (RR), were recorded at five intraoperative time points. Fentanyl was administered if a 20% increase in HR or RR was observed. Results showed that the HR and RR were significantly higher in the control group compared to the QLB group during ovarian manipulation. However, no significant differences were found in systolic or mean arterial pressure between the two groups, and hypotension rates were similar. Importantly, the QLB group required significantly less intraoperative fentanyl compared to the C group. A bilateral QLB with bupivacaine effectively reduces opioid consumption in the study population of cats undergoing elective ovariectomy. This technique may improve pain management and reduce opioids during surgery.

## 1. Introduction

Multimodal analgesic regimes are recommended to optimize perioperative pain control in animals [1,2]. In line with this approach, the use of local anesthetics has been described and proven effective as part of multimodal analgesic protocols in cats undergoing neutering [1,2,3,4,5,6,7].

In recent years, interfascial plane blocks have garnered significant interest as a central strategy for perioperative pain management in human and veterinary medicine [5,6,7,8,9,10]. These novel regional anesthetic techniques have shown efficacy in controlling intraoperative nociception and contributing to a comfortable, pain-free recovery period [5,6,7,8,9,10]. In cats, ultrasound-guided regional anesthetic techniques have been proposed to offer additional perioperative pain control following ovariectomy [5,6,7,11].

The quadratus lumborum block (QLB) is an interfascial plane block aimed to desensitize the sympathetic trunk and thoracolumbar spinal nerves. It involves the deposition of a local anesthetic solution in the interfascial plane between the quadratus lumborum and psoas muscles [12,13]. The QLB appears to offer an adequate level of analgesia for abdominal procedures in several species [5,6,7,8,9,14,15,16]. In cats undergoing ovariectomy, the QLB has been shown to reduce opioid administration, the need for intraoperative rescue analgesia, and the postoperative pain scores [5,6,7]. This technique has also been applied in a cat undergoing cystotomy [14]. Nevertheless, although studies comparing the analgesic efficacy of QLB to a control group without regional anesthesia already exist [7], the specific evaluation of QLB efficacy in cats at four well-defined intraoperative nociceptive stimulation time points has not been investigated yet.

This study aimed to evaluate the effect of a bilateral ultrasound-guided QLB using 0.4 mL kg^−1^ of 0.25% bupivacaine per hemiabdomen on the cats’ intraoperative fentanyl requirements to blunt the increase in heart rate (HR) and/or respiratory rate (RR) to surgical stimuli during ovariectomy in four intraoperative nociceptive times. We hypothesized that compared to a control group (i.e., without QLB), the bilateral ultrasound-guided QLB using 0.4 mL kg^−1^ of 0.25% bupivacaine per hemiabdomen would reduce intraoperative fentanyl requirements in cats undergoing ovariectomy.

## 2. Materials and Methods

### 2.1. Animals

We conducted an a priori sample size estimation using G*Power 3.1.9.7. We determined a minimum sample size of 32 cats (16 per group), considering an estimated effect size of 0.5 (based on our previous group experience) for the reduction in intraoperative opioid administration. This calculation utilized an alpha error of 0.05 and a power of 0.8.

After obtaining approval from the Institutional Animal Care and Use Committee of the Lusofona University—CEBEA (protocol no. 118/2022) and securing consent from a responsible person, 36 healthy feral female cats were enrolled in a prospective, randomized, blinded clinical trial.

All cats were captured one day before the procedure and transported to the hospital in individual cages on the following day. Transport and manipulation of animals were performed with a blanket in accordance with current guidelines [17]. A physical examination, serum biochemistry, and hematology tests were performed on all cats under sedation. Body weight and body condition scores (BCSs) ranging from 1 to 9 were recorded for each cat. Only cats classified as American Society of Anesthesiologists’ status I were included in the study. Food was withheld for at least 6 h, while water was only removed during transport. All animals underwent elective ovariectomy through a median laparotomy. The cats were placed in dorsal recumbency, and a median laparotomy was performed caudal to the umbilicus. The uterine horn was located by digital palpation. After ligating the ovarian pedicle, two clamps were applied to the proximal uterine horn near the ovary, and a ligature was placed distally. The procedure was then repeated on the opposite ovary. The ventral linea alba and subcutaneous tissue were closed with absorbable suture material in a simple, continuous pattern. The skin was closed with an absorbable intradermal suture [18]. Two experienced surgeons, who were also blinded to the study, performed the ovariectomy surgeries.

### 2.2. Group Allocation

**Figure 1:**
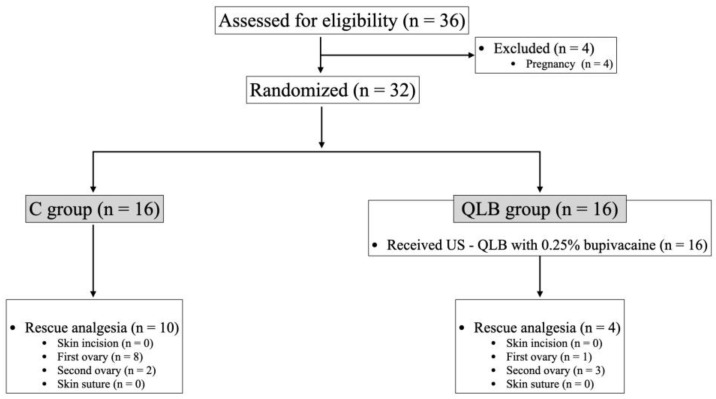
Consolidated standards of reporting trials flowchart describing patient progress through the study. C: control group; *n*: number; QLB: quadratus lumborum block group; US: ultrasound-guided.

Using a random sequence generator (https://www.random.org—accessed on 13 January 2022), cats were randomly assigned to group C (control group; without QLB)) or group QLB (treatment group; with QLB). Cats in group C received intramuscular dexmedetomidine and methadone, whereas cats in group QLB received dexmedetomidine and methadone intramuscularly plus a bilateral ultrasound-guided QLB with 0.4 mL kg^−1^ of 0.25% bupivacaine (Bupivacaine 2.5 mg kg^−1^; B. Braun Medical Inc., Melsungen, Germany) per hemiabdomen (Figure 1). The random sequence results were recorded, and each cat was assigned a number. In order to keep the study blind, there were two anesthetists. One anesthetist (JDS) had access to the list of animals and performed the blocks when appropriate. A second anesthetist (LR), unaware of group allocation, monitored the anesthesia and controlled the need for rescue analgesia. The corresponding group number was only accessible to the anesthetist who performed the block (JDS) and the nurse anesthetist who assisted in the cats’ care.

### 2.3. Anesthesia and Monitoring

Each cat was premedicated intramuscularly with dexmedetomidine (20 μg kg^−1^; Dexdomitor 0.5 mg mL^−1^, Orion, Espoo, Finland) and methadone (0.2 mg/kg; Semfortan 10 mg mL^−1^, Dechra, Italy). A cephalic vein was catheterized under aseptic conditions for drug and fluid administration. Lactated Ringer’s solution (Lactated RingerVet; B.Braun Medical Inc., Melsungen, Germany) was administered intravenously at a rate of 3–5 mL kg^−1^ h^−1^ during the perioperative period. The hair from the sternum to the groin up to the pre-lumbar regions was clipped, and the skin was scrubbed and aseptically prepared. Fifteen minutes after premedication and following two minutes of preoxygenation, general anesthesia was induced with an intravenous injection of 1 to 4 mg kg^−1^ of propofol (Propofol 10 mg mL^−1^; B. Braun Medical Inc., Melsungen, Germany) until loss of palpebral reflex and jaw tone. Cats’ tracheas were intubated with a cuffed endotracheal tube sized 3.5 to 4.5 mm (InTube^®^, Intersurgical, Wokingham, United Kingdom) after applying 0.1 mL of 2% lidocaine (Lidocaine 20 mg mL^−1^, B. Braun Medical Inc., Melsungen, Germany) to the larynx. Anesthesia was maintained with isoflurane (IsoFlo; Zoetis, Madrid, Spain) in oxygen delivered through a non-rebreathing system using a fresh gas flow of 250 mL kg^−1^ min^−1^. The delivered inhalant anesthetic agent concentration (vaporizer dial; Fi’Iso) was averaged for isoflurane in cats. The value for Fi’Iso was selected between 0.8 and 1% based on previous publications [19].

The cats were allowed to breathe spontaneously during surgery. A heating blanket (Thermal Blanket Carbonvet cage, B. Braun Medical Inc., Shanghai China) was used throughout the procedure. After sedation, monitoring was initiated and continued throughout the procedure. Monitoring included continuous electrocardiogram (ECG) and heart rate (HR), respiratory rate (RR; breaths per minute), end-tidal carbon dioxide (Pe’CO_2_; mmHg and kPa), Fi’Iso (%), oesophageal temperature (°C), peripheral hemoglobin oxygen saturation (SpO_2_; %), and heart rate (HR; beats per minute) (BeneVision N15; Mindray, Shenzhen, China). Non-invasive systolic, diastolic, and mean arterial blood pressure (SAP, DAP, and MAP, respectively) were measured every 2 min using a size 2 cuff positioned on either the right or left antebrachium (BeneVision N15; Mindray, Shenzhen, China).

Hypotension was defined as SAP < 90 mmHg or MAP < 60 mmHg. The DAP values were not employed for therapeutic decision-making. The Fi’Iso was reduced by 20% during episodes of hypotension. If reducing isoflurane concentration was not feasible or did not effectively address the hypotension, a 5 mL kg^−1^ bolus of lactated Ringer’s solution was administered over 10 min. If the animal was still hypotensive, a titrated infusion of noradrenaline (0.05 to 0.1 µg kg^−1^ min^−1^) was administered.

### 2.4. US-Guided QL Injection

Shortly after induction of general anesthesia, cats in the QLB group were positioned in right lateral recumbency. An anesthetist (JDS) performed the technique using a 3–13 MHz linear array probe (L12–4s; Mindray, Shenzhen, China) connected to a portable ultrasound machine (TE7; Mindray, Shenzhen, China), following a previously described approach by Dos-Santos et al., 2021 [12]. In brief, the ultrasound transducer was placed perpendicular to the longitudinal axis of the spine at the level of the second lumbar vertebra (L2). The transverse process of L2, quadratus lumborum, psoas minor, and abdominal wall muscles were used as visualization landmarks. A 22 G, 75 mm spinal needle (Spinocan; BBraun Medical, Inc., Melsungen, Germany) was introduced ventrodorsally using an in-plane approach and advanced until its tip reached the interfascial plane between the quadratus lumborum and psoas minor muscles. Following negative aspiration, 0.4 mL kg^−1^ of 0.25% bupivacaine contained in a pre-filled syringe was administered aseptically. The ultrasound visualization of injectate distribution between the QL and Pm muscles was considered a confirmation of correct administration. Subsequently, the cat was positioned in left lateral recumbency, and the procedure was repeated on the contralateral site. The total execution time, measured from the initial transducer–skin contact to the completion of injection for each hemiabdomen, was recorded. In the C group, no regional anesthetic technique or sham block was performed.

### 2.5. Data Collection

Values of HR, RR, SAP, and MAP were collected at baseline and following four intraoperative time points: skin incision, first (right) ovary manipulation, second (left) ovary manipulation, and skin closure. Baseline values were documented before the first skin incision. The surgery started 15 min after the induction of anesthesia.

Response to surgical stimuli was defined as a >20% increase in HR and/or RR compared to baseline measurements. In the presence of such a response, anesthetic depth was adjusted per the clinical judgment of the anesthetist in charge based on the evaluation of palpebral reflex, jaw tone, or movement. Response to stimuli with adequate anesthetic depth was judged as a nociceptive response, and a bolus of fentanyl (4 μg kg^−1^; Fentadon; 50 µg mL^−1^, Dechra, Turin, Italy) was administered as rescue analgesia. The duration of anesthesia (from induction to extubation) and of the surgical procedure (from skin incision to skin closure) were recorded for analysis.

Statistical analysis was carried out using SPSS version 26 (IBM SPSS Statistics; IBM Corp., New York, NY, USA). Data normality was assessed using the Shapiro–Wilk test. The non-normally distributed variable BCS was described using the median and interquartile range (IQR), and the Mann–Whitney U test was employed for median comparisons. For normally distributed variables, such as demographic data, blood test results, anesthesia time, surgical time, isoflurane vaporizer percentage, and time to extubation, mean and standard deviation (mean ± SD) were presented. For the analysis of the repeated measures of the variables HR, SAP, MAP, and RR, a generalized linear mixed model (GLMM) was used, considering group and time as fixed factors and individual variability between cats as a random effect.

To test the relationship between groups for the variables of rescue analgesia and hypotension, a Fisher’s exact test was applied. Significance was established at *p* < 0.05.

### 2.6. Postoperative Period

After extubation, all cats received meloxicam (0.2 mg kg^−1^; Metacam; 5 mg kg^−1^, Boehringer Ingelheim Vetmedica Gmbh, Ingelheim, Germany) subcutaneously. Postoperative monitoring, which began immediately after extubation, included assessment of HR, RR, oral mucous membrane colour, and temperature. This monitoring was conducted before the cats were placed in individual clean cages for recovery. The animals were discharged 8 h after completion of surgery.

## 3. Results

### 3.1. Demographic

A total of 36 female domestic shorthair cats scheduled for ovariectomy were initially enrolled in the study. Four animals were excluded due to pregnancy, resulting in a final count of 32 cats (*n* = 16 in group C and *n* = 16 in group QLB). Figure 1 presents the flowchart illustrating the enrolment process. In the QLB group, US visualization of landmarks and injection sites were possible in all hemiabdomens. In all cases, visualization of injectate distribution in the target interfascial plane was possible and without complications.

Demographic information, blood test results, as well as the duration of anesthesia and surgery are presented in Table 1. The average time to perform the ultrasound-guided QLB per hemiabdomen was 2.5 ± 0.76 min. Compared to the C group, the execution of the QLB extended the mean anesthesia time by 7.12 (*p* = 0.005; Table 1). The Fi’Iso values were 0.9 ± 0.2% and 1 ± 0.1% for groups QLB and C, respectively (*p* > 0.05).

### 3.2. Monitoring

**Figure 2:**
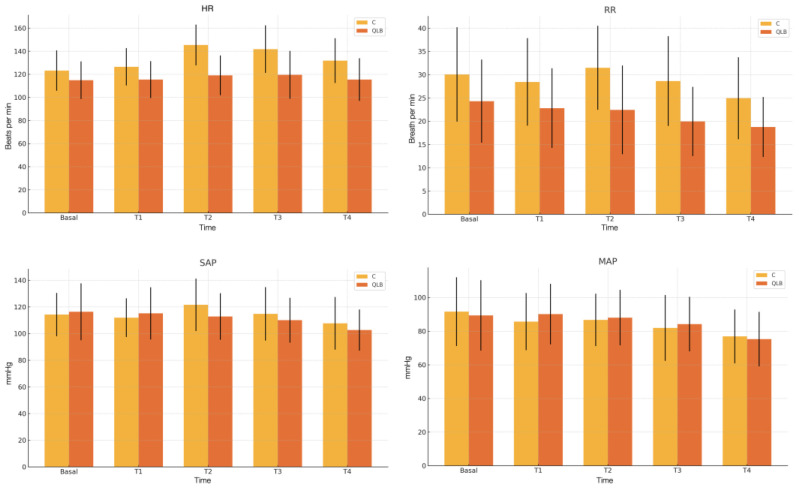
Intraoperative in mean and standard deviation in pulse rate, respiratory rate, systolic arterial pressure, and mean arterial pressure in two treatment groups in five different surgical times. C: control group; MAP: mean arterial pressure; HR: pulse rate; QLB: ultrasound-guided quadratus lumborum block with 0.25%bupivacaine; RR: respiratory rate; SAP: systolic arterial pressure.

The hemodynamic and respiratory variable progression recorded throughout the intraoperative period is presented in Figure 2. Throughout the surgery, both HR and RR were consistently higher in the C group compared to the QLB group in all time points evaluated (*p* < 0.05; Figure 2). However, there were no significant differences in SAP and MAP between the groups (Figure 2). Notably, hypotension was noted in 12.5% (2/16) of cats in the C group and 18.75% (3/16) in group QLB, with no statistically significant difference between groups (*p* > 0.05).

### 3.3. Rescue

Rescue analgesia with fentanyl was required in 62.5% (10/16) of cats in the C group and 25% (4/16) of cats in the QLB group, with a statistically significant difference (*p* < 0.05). In the C group, 80% (8/10) of nociceptive events necessitating rescue analgesia occurred during manipulation of the first ovary, while 20% (2/10) occurred during manipulation of the second ovary. Conversely, in the QLB group, 25% (1/4) of rescue analgesia cases followed manipulation of the first ovary, whereas 75% (3/4) occurred after manipulation of the second ovary.

## 4. Discussion

The present study demonstrated that the use of a bilateral ultrasound-guided QLB with 0.4 mL kg^−1^ of 0.25% bupivacaine, in addition to systemic analgesia (i.e., dexmedetomidine and methadone), resulted in reduced intraoperative fentanyl requirements. These findings support the hypothesis that incorporating this regional anesthetic technique contributes to reducing the nociceptive response during ovariectomy.

Cadaveric studies in cats [12,13] have confirmed that injecting dye between the quadratus lumborum and psoas minor muscles at the level of L2 predictably stains the ventral branches of the last thoracic and first lumbar spinal nerves, together with sympathetic nerve involvement. This pattern of distribution could have accounted for the reduced nociceptive response in the QLB group compared to the C group. In this study, the QLB prevented the nociceptive response in 75% of the cats. Conversely, only 37% of the cats in the C group did not require analgesic rescue. Given that the only treatment difference between the C and QLB groups was the regional anesthetic technique, it is reasonable to propose it as the main determinant of the differing outcomes. These results are consistent with findings from a similar study conducted in cats, in which the use of a QLB effectively prevented a response to surgical stimulation in 80% [5] and 94.5% [6] of the animals included. It is important to note that the cats enrolled in this study had an ideal BCS, which facilitated the ultrasound identification of landmarks and presumably contributed to the successful execution of the blockade when compared with QLB in dogs [20].

In this study, all rescue interventions occurred during the ligation of the ovarian pedicles. Traction and ligation of the ovarian pedicle have been identified as nociceptive critical points during this type of surgery [21,22]. Cadaveric studies on QLB in cats have demonstrated the distribution of dye in the somatic and visceral afferent pathways involved in ovarian innervation [12,13]. As a result, it is possible that the nerve block induced by the QLB contributed to the reduction of nociception associated with ovarian pedicle manipulation in this study. The lack of nociceptive response during the manipulation of ovarian pedicles and the minimal response percentage to the surgical stimulus (as indicated by HR, RR, and the number of animals requiring fentanyl) observed in the QLB group suggest an improvement in nociception control. In the QLB group animals where rescue with fentanyl was necessary, this may have occurred due to suboptimal distribution, anatomical variations, or technical failure by the anesthetist. Our research is in line with other clinical investigations conducted in humans [23,24], dogs [9,21], and cats [5,6,7]. These studies demonstrate that adding a QLB in a multimodal analgesic regime could enhance intraoperative analgesia during various abdominal surgical procedures.

Despite the longer mean anesthesia time observed in the QLB group compared to the C group, the reduction in intraoperative rescue analgesia requirements may justify this additional time. In this study, the chosen volume (0.4 mL/kg) and concentration (0.25% bupivacaine) were based on previous studies in cats [5,6] and commonly applied protocols in other species [9,16,25]. Future studies should investigate the effects of varying volumes and concentrations to determine whether the block’s efficacy can be maintained under different conditions.

In this study, the incidences of hypotension were 18.75% and 12.5% in the QLB and C groups, respectively. The Fi’Iso was the same in both groups. The hypotension observed after the QLB might be attributed to the sympathetic block caused by the local anesthetic and the subsequent vasodilation [26,27]. Furthermore, cadaveric studies of QLB in cats consistently show dye dispersion along the sympathetic trunk, splanchnic nerves, and celiac ganglion pathways [12,13]. The results of this study are consistent with a previous investigation involving cats, where queens that received a QLB displayed more frequent hypotension compared to those without QLB (10% versus 5%; [6]) despite not showing statistically significant differences between groups. Less likely, hypotension resulting from a QLB could also be explained by systemic absorption of the local anesthetic [26,27].

This study has several limitations. The fentanyl dose used for rescue analgesia in this study, while within the reference values for the species, might be reduced in future studies with the aim of decreasing opioid administration. The blood test results were obtained after sedation, which could have some influence on the results. Postoperative data collection was a challenge due to the feral behavior of the cats, impeding the evaluation of the efficacy and potential benefits of the QLB during recovery. Due to the lack of invasive blood pressure monitoring, real-time assessment of the nociceptive response to stimuli was limited to the evaluation of HR and RR. Likewise, the prevalence of hypotension was evaluated by means of non-invasive blood pressure methods. The use of dexmedetomidine at 20 µg/kg might have biased the evaluation of intraoperative nociception, which indicates the necessity for additional research using lower doses or alternative sedation protocols. Lastly, the QLB performed by a single anesthetist may also be a source of bias and calls for future investigations involving multiple operators.

## 5. Conclusions

This study demonstrated that a bilateral ultrasound-guided QLB, using 0.4 mL kg^−1^ of 0.25% bupivacaine per hemiabdomen, in combination with dexmedetomidine and methadone, successfully reduced opioid administration in feral cats undergoing elective ovariectomy. Further clinical investigations are warranted to assess the perioperative benefits and the complications associated with this technique.

## Figures and Tables

**Table 1:** Mean ± standard deviation of demographic data, blood test results, anesthesia time, surgical time, and time to extubation in cats undergoing ovariectomy. The body condition score variable data are presented as median (interquartile range; [IQR]). Significance was set at *p* < 0.05 (*). C: control group; BCS: body condition score; *n*: number; QLB: ultrasound-guided quadratus lumborum block group with 0.25% bupivacaine.

Variable	C (*n* = 16)	QLB (*n* = 16)	*p* Value
Body weight (kg)	2.86 ± 0.64	3.29 ± 0.58	0.150
BCS (1–9) (median [IQR])	4 [1.75]	4 [1.75]	0.582
Hematocrit (%)	45.00 ± 10.73	37.12 ± 5.48	0.012 *
Blood glucose (mg/dL)	112.38 ± 35.16	203.06 ± 62.40	0.000 *
Total protein (g/dL)	6.88 ± 0.53	7.55 ± 1.01	0.363
Platelets (×10^3^/µL)	296.81 ± 93.54	300.31 ± 127.82	0.882
Anesthesia time (min)	54.4 ± 7.9	61.6 ± 5.1	0.005 *
Surgical time (min)	23.5 ± 4.9	20.3 ± 5.9	0.207
Time to extubation (min)	9.88 ± 4.62	11.00 ± 4.03	0.571

## Data Availability

Data are contained within the article.

## References

[1] Gurney M.A. (2012). Pharmacological options for intra-operative and early postoperative analgesia: An update. J. Small Anim. Pract..

[2] Steagall P.V., Robertson S., Simon B., Warne L.N., Shilo-Benjamini Y., Taylor S. (2022). ISFM Consensus Guidelines on the Management of Acute Pain in Cats. J. Feline Med. Surg..

[3] Benito J., Monteiro B., Lavoie A.-M., Beauchamp G., Lascelles B.D.X., Steagall P.V. (2016). Analgesic efficacy of intraperitoneal administration of bupivacaine in cats. J. Feline Med. Surg..

[4] Garbin M., Ruel H.L., Watanabe R., Malo A., Monteiro B.P., Steagall P.V. (2023). Analgesic efficacy of an ultrasound-guided transversus abdominis plane block with bupivacaine in cats: A randomised, prospective, masked, placebo-controlled clinical trial. J. Feline Med. Surg..

[5] Dos-Santos J.D., Ginja M., Martins J., Cabral P., Alves-Pimenta S., Ribeiro L., Otero P.E., Colaço B. (2024). Comparison between Bilateral Ultrasound-Guided Quadratus Lumborum Block and Sacrococcygeal Epidural in Cats Undergoing Ovariectomy. Vet. Sci..

[6] Paolini A., Bianchi A., Bucci R., Parrillo S., Di Giosia A., Ristori C., Carluccio A., Tamburro R., Vignoli M., Collivignarelli F. (2024). Use of a quadratus lumborum block in queens undergoing ovariectomy: A randomised controlled trial. J. Feline Med. Surg..

[7] Lazzarini E., Gioeni D., Del Prete G., Baio M., Carotenuto A.M. (2024). Ultrasound-guided quadratus lumborum block with 0.5 mL of 0.2% bupivacaine/kg is a valuable perioperative analgesic adjunct for cats undergoing ovariectomy. J. Am. Vet. Med. Assoc..

[8] Elsharkawy H., Pawa A., Mariano E.R. (2018). Interfascial plane blocks: Back to basics. Reg. Anesth. Pain Med..

[9] Viscasillas J., Sanchis-Mora S., Burillo P., Esteve V., Del Romero A., Lafuente P., Redondo J.I. (2021). Evaluation of quadratus lumborum block as part of an opioid-free anaesthesia for canine ovariohysterectomy. Animals.

[10] Viilmann I., Drozdzynska M., Vettorato E. (2022). Analgesic efficacy of a bilateral erector spinae plane block versus a fentanyl constant rate infusion in dogs undergoing hemilaminectomy: A retrospective cohort study. BMC Vet. Res..

[11] Skouropoulou D., Lacitignola L., Centonze P., Simone A., Crovace A.M., Staffieri F. (2018). Perioperative analgesic effects of an ultrasound-guided transversus abdominis plane block with a mixture of bupivacaine and lidocaine in cats undergoing ovariectomy. Vet. Anaesth. Analg..

[12] Dos-Santos J.D., Ginja M., Alves-Pimenta S., Otero P.E., Ribeiro L., Colaço B. (2021). A description of an ultrasound-guided technique for a quadratus lumborum block in the cat: A cadaver study. Vet. Anaesth. Analg..

[13] Dos-Santos J.D., Ginja M., Alves-Pimenta S., Otero P.E., Ribeiro L., Colaço B. (2022). Comparison of dorsoventral and ventrodorsal approaches for ultrasound-guided quadratus lumborum block in cats: A cadaver study. Vet. Anaesth. Analg..

[14] Argus A.P., Freitag F.A., Bassetto J.E., Vilani R.G. (2020). Quadratus lumbar block for intraoperative and postoperative analgesia in a cat. Vet. Anaesth. Analg..

[15] Klonner M.E., Verdier N., Otero P.E. (2023). Bilateral ultrasound-guided lateral quadratus lumborum block in a minipig undergoing ovariectomy. Vet. Rec. Case Rep..

[16] Vettorato E., Schmidt K.J., Horgan M.D., Chiavaccini L., Portela D.A. (2023). Quadratus lumborum block as part of multimodal analgesia in a rabbit undergoing liver lobectomy. Vet. Anaesth. Analg..

[17] Rodan I., Dowgray N., Carney H.C., Carozza E., Ellis S.L., Heath S., Niel L., Denis K.S., Taylor S. (2022). AAFP/ISFM Cat Friendly Veterinary Interaction Guidelines: Approach and Handling Techniques. J. Feline Med. Surg..

[18] Langley-Hobbs S.J., Demetriou J., Ladlow J.F. (2014). Feline Soft Tissue and General Surgery.

[19] Steffey E.P., Howland D. (1977). Isoflurane potency in the dog and cat. Am. J. Vet. Res..

[20] Garbin M., Portela D.A., Bertolizio G., Garcia-Pereira F., Gallastegui A., Otero P.E. (2019). Description of ultrasound–guided quadratus lumborum block technique and evaluation of injectate spread in canine cadavers. Vet. Anaesth. Analg..

[21] Vicente D., Bergström A. (2018). Evaluation of intraoperative analgesia provided by incisional lidocaine and bupivacaine in cats undergoing ovariohysterectomy. J. Feline Med. Surg..

[22] Carapeba G.d.O., Nicácio I.P.G.A., Stelle A.B.F., Bruno T.S., Nicácio G.M., Júnior J.S.C., Giuffrida R., Neto F.J.T., Cassu R.N. (2020). Comparison of perioperative analgesia using the infiltration of the surgical site with ropivacaine alone and in combination with meloxicam in cats undergoing ovariohysterectomy. BMC Vet. Res..

[23] Blanco R., Ansari T., Riad W., Shetty N. (2016). Quadratus lumborum block versus transversus abdominis plane block for postoperative pain after cesarean delivery: A randomized controlled trial. Reg. Anesth. Pain Med..

[24] Blanco R., Ansari T., Girgis E. (2015). Quadratus lumborum block for postoperative pain after caesarean section: A randomised controlled trial. Eur. J. Anaesthesiol..

[25] Ribeiro C., Dos-Santos J.D. (2023). Opioid-free anaesthesia for surgical attenuation of a congenital extrahepatic portosystemic shunt in a dog. Vet. Rec. Case Rep..

[26] Sá M., Cardoso J.M., Reis H., Esteves M., Sampaio J., Gouveia I., Carballada P., Pinheiro C., Machado D. (2018). Bloqueio do quadrado lombar: Estamos cientes de seus efeitos colaterais? Relato de dois casos. Braz. J. Anesthesiol..

[27] Chadwick H.S. (1985). Toxicity and resuscitation in lidocaine or bupivacaine-infused cats. Anesthesiology.

